# Working Women in Large Towns

**Published:** 1890-09-27

**Authors:** 


### Working Women in Large Towns.

### XI.—SHOP ASSISTANTS.

We are sometimes tempted to fear that this is an age of words rather than deeds—of commissions, meetings, debates, and so forth; and in the end, nothing to show for them. Much has been written, much has been said, about the unfortunate shop-assistant; but nothing, or almost nothing, has been done. The Early Closing Bill has been thrown out of the House of Commons, and the Weekly Half Holiday Bill, blocked by Mr. Blundell Maple, seems to have but a poor chance of a speedy hearing.

But is legislation really necessary? Let us state the case. In London, and in our large towns, many thousands of young girls stand behind the counter from thirteen to seventeen hours in the day, with but a short break—twenty minutes, perhaps—for dinner, and another fifteen minutes, for tea. They have no time for recreation, nor for improving their minds; no chance to get a breath of fresh air, except by stealing an hour from their much-needed rest, and the Saturday half-holiday, looked forward to by so many workers, simply means to them an extra two or three hours of labour. When they sleep on the premises, they are often crowded into small and unhealthy rooms, their food is very often ill-cooked and poor in quality, and the scanty time allowed for eating it is apt to be broken in upon by calls to the counter. The long hours of standing bring on various kinds of illness, as well as painful swelling of the feet and legs; and large numbers of shop assistants have to leave their situations utterly broken down in health.

Many of them begin work at twelve or thirteen; indeed, we hear that from one-half to two-thirds of the shop assistants of this country are boys and girls from twelve to twenty-one years of age. A frightful strain is thus put upon them just at the time they are least able to stand it; they struggle on for a few years, losing health and strength and all the joyous elasticity of youth; and then they drop out, and go back to their homes—if homes they have—to pine and languish, perhaps to die. What matter? There are plenty of others ready enough to take their place. No need to fear that the public will not be served; it has lived upon the only capital of those young girls—their health and strength; and it will have plenty of chances of doing the like again.

Are we speaking too strongly? Hear what the doctors say. "It is impossible," writes Dr. Norman Kerr, consulting physician to an Inebriates' Home, "for me to find language strong enough to convey a hundredth part of the mischief which I have seen arise from the excessive hours of labour of shop assistants." Another doctor, medical officer of an insurance company, tells us that "shop-assistants and clerks give the largest percentage of phthisis, showing incontestably that the employment in shops is a fruitful source of that terrible disease, the influence of which descends to a succeeding generation." In fact, doctors are unanimous as to the terrible harm wrought, more especially upon women, by long hours of standing; and if any corroboration of their testimony is needed, it may be found in the simple statements of the girls themselves. One after another will tell you of the ill-health and lassitude of herself and of her companions.

"The doctor says I am in a consumption, and I shall have to leave at the end of my month," says one. "I scarcely know," says another, "what it is to stand with ease for the violent pains in my feet and legs, and more often in my back. My feelings at the end of the day are so dreadfully low and weak that I scarcely have the strength to undress."

These witnesses, as Mr. Sutherst, President of the Shop Hours Reform League, truly observes, could be corroborated by thousands more. One after another tells the same sad tale of broken-down health and flagging energies; of fruitless longings for fresh air, and for time to attend classes and lectures; of companions or sisters who have left work only to die; of Sundays, when "my only thought is to rest." And if any further confirmation of the truth of their statements is needed, it may surely be found in the fact that the Shop Hours Reform League, supported by Cardinal Manning, and by the late Archbishop of Canterbury, has been obliged to hold its meetings on a Sunday, it being found impossible for the shop assistants to attend them on any other day. We do not believe that any workers in England, unless it may be some home-workers—and they are, after all, their own masters and mistresses—are kept so long on the grind as these unfortunate shop assistants.

True, it is not all shop assistants who are in this miserable plight. In fashionable West-end shops, the hours are reasonable, and the various conditions of the shop assistants' life far less trying, sometimes indeed, all, or nearly all, that could be wished. And this, no doubt, accounts for, and in part excuses, the ignorance and apathy of the public with regard to this matter. It is in the smaller shops, and the poorer neighbourhoods, such as Islington, Peckham, New Cross, Battersea, &c., &c., that the greater part of the mischief is done. And the shopkeepers themselves are not much to blame; indeed, many of them cry out for shorter hours themselves, and would be only too thankful if they could close earlier without risk of losing their business. They must indeed keep their shops open later than do fashionable shopkeepers, but the poor need not shop as late as they do, and, at any rate, a weekly half-holiday would do no harm to them, and incalculable good to the shop-keeper and his assistants. Efforts have of late years been made, as is well known, to secure this weekly half-holiday by means of combination among the shop-keepers, and in some towns these efforts have been successful, but we fear that very much will never be done in this way. In place after place we hear the same story: the weekly half-holiday was given for a year or two, but was then dropped, owing to the selfishness and meanness of some one shop-keeper, who promised to do as others did, and then backed out. Or again, a new shop-keeper may at any time appear on the scenes, bound by no promise, and only anxious to seize the advantage thus offered to him. And as for the idea that the feelings of the public might be worked upon so that they would voluntarily forego all late shopping, that might be possible (though we doubt it) were the upper classes in question, but is manifestly hopeless, considering that it is the poor, and not the rich, who do the greater part of the mischief.

Have we made out a case for legislation? We think we have. While fully acknowledging that legislation should never be resorted to until other means have been shown to fail, we see no reason why the law should not step in in this case, and many reasons why it should. We do not think it has ever been asserted that the work which is now spread out over thirteen, fourteen, and even seventeen hours a-day could not be done, and equally well done, in less; or that a weekly half-holiday would do any injury to trade. And if the law steps in to protect children and women working in factories, why should it not also protect them when working in shops? Can it be said that children of twelve or fourteen are able to look after themselves—to strike for shorter hours and better conditions of labour? All reason and all experience are against it. And are we to leave them to their fate? to watch unmoved the working of a system which, as Mr. Sutherst puts it, "consumes the ripening energies of healthy young persons, and then throws them off as worthless, only to devour a fresh supply?"

To say nothing of the cruelty of such a process, can we afford to let it go on? Are we prepared to let the mothers of the future generation ruin their constitutions, and hand down diseased and enfeebled frames to the children they bear?

